# The effects of adding epinephrine to ropivacaine for popliteal nerve block on the duration of postoperative analgesia: a randomized controlled trial

**DOI:** 10.1186/s12871-015-0083-z

**Published:** 2015-07-10

**Authors:** Karin P.W. Schoenmakers, Maaike G.E. Fenten, Jan Willem Louwerens, Gert Jan Scheffer, Rudolf Stienstra

**Affiliations:** 1 Department of Anaesthesiology, Post box 9011, 6500 GM Nijmegen, The Netherlands; 2 Orthopaedic Surgery Sint Maartenskliniek, Post box 9011, 6500 GM Nijmegen, The Netherlands; 3 Department of Anaesthesiology, Radboud University Medical Center, Nijmegen, The Netherlands

**Keywords:** Peripheral nerve block, Local anesthetic adjuncts, Postoperative analgesia

## Abstract

**Background:**

Duration of peripheral nerve blocks depends on multiple factors. Both technique and type of local anesthetic used, either with or without adjuncts, may result in different duration times of the block. The purpose of the present study was to compare the duration of postoperative analgesia of 30 mL ropivacaine 0.75 % with or without epinephrine for popliteal sciatic nerve block.

**Methods:**

Thirty-eight patients were included to receive ultrasound guided continuous popliteal nerve block with ropivacaine 0.75 % either without (ROPI) or with epinephrine 5 μg/mL (ROPI-EPI) for ankle fusion, subtalar fusion, or a combination of both. The primary outcome parameter was the duration of postoperative analgesia as reflected by the time to first request for postoperative analgesia (TTFR) through the popliteal nerve catheter. Secondary outcome measures included the onset of sensory and motor block and NRS score for pain at rest and during movement.

**Results:**

Thirty patients, 15 in each group, were studied. Eight patients were withdrawn because of specific withdrawal criteria described in the protocol and replaced according to their group allocation. Median [interquartile range] TTFR was 463 [300–1197] min and 830 [397–1128] min for the ROPI vs ROPI-EPI group respectively. Hodges Lehman median difference between groups was 71 min (95 % CI −415 – 473 min). There was no difference in any clinical outcome measure between the groups.

**Conclusion:**

The results of this study did not show any significant increase in the duration of postoperative analgesia by adding epinephrine to ropivacaine for popliteal nerve block. This may be due to the intrinsic vasoconstrictive properties of ropivacaine. The absence of a significant difference can also be the result of a type II error caused by a large variation in the individual TTFR.

**Trial registration:**

Trial register.nl identifier: NTR3330, keyword TTFR

## Background

Duration of peripheral nerve block depends on several factors such as the choice of local anaesthetic (LA), the site of injection and the presence of adjuncts such as clonidine or epinephrine. Epinephrine may be added to (large) doses of local anaesthetics with the objective to reduce the maximum plasma concentration [1] or to act as a marker for inadvertent intravascular injection [2]. The rationale for adding epinephrine to reduce the maximum plasma concentration is local vasoconstriction at the site of injection [3], thereby slowing absorption. A decrease in absorption increases the duration of analgesia [4]. The literature however, is inconclusive regarding this effect. Several studies find a decrease in C_max_ and an increase in t_max_ as result of adding epinephrine to ropivacaine for epidural [5, 6], caudal [7] or regional [8] (thoracic paravertebral block) anaesthesia confirming a decrease in absorption. However, others fail to confirm prolonged sensory block duration when adding epinephrine to ropivacaine [9, 10]. In a recent study aimed to describe the pharmacokinetic profile of high dose ropivacaine with and without epinephrine [11], we found an indication of prolonged time to first request for postoperative analgesia (TTFR) after the addition of epinephrine to ropivacaine for combined sciatic/femoral nerve block for anterior cruciate ligament reconstruction. In this study of 12 patients [11], 3 did not request postoperative analgesia (1 in the ROPI-group and 2 in the ROPI-EPI-group). For the remaining patients the median TTFR was 17 [12.5-22] h in the ROPI-EPI-group and 3.5 [3–17] h in the ROPI-group. Because of the small number of patients, these data have not been included in the original publication.

The purpose of the present study is to compare the duration of postoperative analgesia of 30 mL ropivacaine 0.75 % with or without epinephrine for popliteal sciatic nerve block.

## Methods

### Patients

This prospective double blinded (for observer and patient) randomized study was approved by the Independent Review Board Nijmegen (protocol number NL39628.072.12, date of approval 28-02-2012) and was registered at http://www.trialregister.nl (NTR3330, keyword TTFR) before onset of participant enrolment. The study was conducted at the Sint Maartenskliniek Nijmegen, The Netherlands according to the Declaration of Helsinki and later revisions thereof and in accordance with the ICH guidelines for Good Clinical Practice.

Patients scheduled for continuous popliteal sciatic nerve block for ankle fusion, subtalar fusion, or a combination of both were assessed for eligibility during the preoperative screening visit. Patients were informed about the study verbally and in writing and written informed consent was obtained from all patients. Eligible participants were all adults aged 18 or over with ASA physical health classification I-III. Exclusion criteria included contra-indications for regional anaesthesia (infection at the injection site, coagulopathy), known hypersensitivity to amide-type local anaesthetics, known history of peripheral neuropathy, inability to understand numerical pain scales, and inability to operate a Patient-Controlled Analgesia (PCA) device. Specific criteria for withdrawal (and replacement) included: failure to perform adequate continuous popliteal sciatic nerve block; pain in the distribution of the sciatic nerve upon arrival at the recovery directly postoperatively requiring a therapeutic intervention (block failure); and failure to complete the study protocol (e.g. no request for postoperative analgesia).

### Anaesthetic procedure

All patients received paracetamol 1000 mg orally three times daily and etoricoxib 90 mg orally once a day, starting on the morning of surgery for at least 7 days. Intravenous access and routine monitoring were established in all patients. Individuals were randomised by a computer-generated random list in blocks of five and were concealed in sequentially numbered, sealed, opaque envelopes prepared by an independent investigator. The envelopes were opened by an independent anesthetic nurse after participant details were written on the envelope. An assignment card inside the envelope read either “30 mL ropivacaine 0.75 % without 5 μg/mL epinephrine” or “30 mL ropivacaine 0.75 % with 5 μg/mL epinephrine”. Accordingly, patients received continuous popliteal nerve block with ropivacaine either without (ROPI, n = 15) or with epinephrine (ROPI-EPI, n = 15). Study medication was disclosed to the anesthesiologist performing the block procedure. All popliteal blocks were placed with the patient in the lateral decubitus position on the non-dependent side using ultrasound guidance and a posterolateral in-plane approach. A nerve stimulator set to deliver 100 nC (1 mA, 0.1 ms) at 2 Hz was used as an additional aid. The tibial, peroneal and sciatic nerves were identified and injection was made at the level of the bifurcation of the sciatic nerve. Thirty mL ropivacaine 0.75 % without or with epinephrine 5 μg/mL was injected in fractionated doses. Time was designated t = 0 upon conclusion of the popliteal sciatic nerve block. A perineural catheter was inserted through the needle after injection of the loading dose. Upon completion of the popliteal nerve block the patient was placed in the supine horizontal position. Because surgery was performed under exsanguination and a tourniquet, a single shot ultrasound-guided femoral or saphenous nerve block with 20 mL mepivacaine was performed to facilitate the use of the tourniquet. Surgery was performed under regional anaesthesia alone, or supplemented with sedation upon patient request. If the planned duration of surgery exceeded 120 min, patients received general anaesthesia with propofol, remifentanil and a laryngeal mask.

Upon arrival at the recovery room, a PCA-pump (GemStar®, Hospira Inc. Lake Forest, Illinois, USA) was connected to the popliteal catheter set up to deliver bolus doses of 10 mL ropivacaine 0.2 %, with a lock-out time of 15 min, no background infusion and a maximum of 30 mL per 4 h. The intensity of postoperative pain was evaluated by Numeric Rating Scale (NRS, 0–10 with 0 representing no pain and 10 worst possible pain). Prior to surgery, patients had been instructed to use the PCA device to maintain postoperative pain scores at or below NRS 3. No regular postoperative opioids were provided. If patients still needed pain medication after their first request for ropivacaine through the popliteal catheter (ie after primary endpoint of the study), patients received morphine 0.1–0.15 mg/kg every 4 h subcutaneously.

### Clinical assessments

Baseline characteristics of participating patients were recorded (i.e. age, length and weight).

During the first 45 min after performance of the popliteal nerve block, a blinded observer assessed the onset of sensory and motor block every 5 min until complete block of the tibial and peroneal nerve. Sensory block of the tibial and peroneal nerves was assessed by pinprick at the heel of the foot (tibial nerve) and dorsum of the foot between the 1st and 2nd toe (peroneal nerve). Sensory block was scored on a three-point scale as 0 = absent, 1 = partial and 2 = complete. Motor function of the tibial (plantar flexion foot) and peroneal nerve (dorsal flexion of foot) were also assessed on a three-point scale with 0 = no motor block, 1 = partial and 2 = complete motor block. Complete sensory and motor block was defined as a total score of 8. In those patients that did not have a complete block before the beginning of surgery, we assessed block success upon arrival at the recovery room directly postoperatively. In these patients, block success was defined as absence of pain requiring therapy in the distribution of the sciatic nerve distribution area upon arrival at the recovery room, whereas patients requiring pain relief at this time were deemed failures and were excluded from the study. Type and duration of surgery were recorded.

Because of the expected long duration of the sciatic nerve block with ropivacaine, measuring the offset of sensory and motor block similar to measuring the onset would have implied frequent measurements during the night. We considered this both unethical and impractical, therefore we did not measure offset, but chose the TTFR as the primary outcome parameter reflecting the duration of sensory sciatic nerve block/duration of analgesia. At t = 24 h the PCA pump was read out and the TTFR was noted. TTFR was defined as the time from t = 0 until the time that the patient made the first request for analgesia via the PCA pump. In case no request had been made, the presence of sensory and/or motor block were evaluated in the same manner as preoperatively. If at this time patients who had made no request had no signs of sensory sciatic nerve block, they were excluded and replaced because of absence of the primary outcome parameter. In case patients still showed signs of partial or complete sciatic nerve block, the observation period was extended to t = 48 h.

The primary outcome parameter was the duration of postoperative analgesia as reflected by the TTFR. Secondary outcome measures included the onset of sensory and motor block, NRS score for pain at rest and during movement directly postoperatively, at t = 24 h and if necessary at t = 48 h and satisfaction score (NRS 0–10) with the anaesthetic technique at the time of completion of the study.

### Sample size and statistical analysis

Taboada et al. [12] have reported a duration of 19 ± 3.4 h postoperative analgesia after popliteal block with 30 mL ropivacaine 0.75 %. Based on these data, the sample size required to have an 80 % probability of detecting a difference of 20 % (two-sided, level of significance 0.05) in the duration of postoperative analgesia between the groups was 12 patients per group. We chose to include 15 patients per group to compensate for variation in standard deviation. Patients with specific withdrawal criteria, as mentioned earlier in the methods section, were withdrawn and replaced. An independent monitor not involved in further conduction of the study made new sealed envelopes according to group allocation of the withdrawn patients.

Data were analysed using the GraphPad Prism 6 software (GraphPad Software Inc, San Diego, CA). Analysis was per protocol. The D’Agostino & Pearson omnibus normality test was used for normality testing. Continuous, normally distributed data are presented as mean ± SD [range], non-normally distributed data as median [interquartile range]. For statistical comparison between the groups, the student-*t* test for normally distributed data and the Mann Whitney *U* test for nonparametric comparisons was used. In case a parameter is normally distributed in one group and non-normally in the other group, the data are presented as median [interquartile range] and a nonparametric test used for statistical comparison. The Hodges-Lehmann estimate was used for calculating the difference between population medians with 95 % CI; the difference between each value in the ROPI group and each value in the ROPI-EPI group was computed and the Hodges-Lehmann estimate is the median of this set of differences. The part of patients without need for postoperative analgesia in group ROPI vs ROPI-EPI were compared using Chi squared test. A *p*-value < 0.05 was considered statistically significant.

## Results

**Fig. 1:**
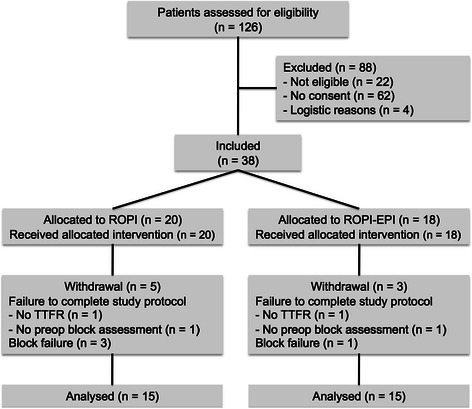
Consort flowchart of patients enrolled in the study

In order to study 15 patients in each group, 38 patients were included in the study protocol between July 2012 and March 2013. A Consort flowchart is shown in Fig. 1. Five patients in the ROPI group and three in the ROPI-EPI group were withdrawn on account of specific withdrawal criteria described in the protocol. These included absence of the primary outcome parameter: no request for postoperative analgesia at t = 48 h and absence of sensory sciatic nerve block (one in each group); pain requiring therapy in the distribution of the sciatic nerve upon arrival at the recovery directly postoperatively (block failure; three in the ROPI group, one in the ROPI-EPI group); and start of surgery before preoperative block assessment could be made (one in each group). The latter two patients were withdrawn from the study because there was no time for the sciatic nerve block to develop and the attending anesthesiologists were allowed to use long acting opioids at their own descretion. There were no significant differences in patient characteristics between the two groups (Table 1). None of the patients showed signs of local anaesthetic systemic toxicity or inadvertent intravascular injection of epinephrine (such as rise in heart rate, systolic blood pressure or flushing).

**Table 1:** Patient characteristics

	ROPI	ROPI-EPI
Sex (M/F)	8/7	9/6
Age (yr)	61 ± 7	56 ± 11
Length (cm)	177 ± 12	175 ± 8
BMI (kg/m^2^)	28 ± 4	30 ± 5
ASA (I/II/III)	4/10/1	5/10/0
Surgery (*)	Ankle fusion: 9 (1)	Ankle fusion: 10 (3)
	Subtalar fusion: 6	Subtalar fusion: 4 (1)
	Combination: 0	Combination: 1
Operation time (min)	87 ± 33	92 ± 33

*ROPI* popliteal block with 30 mL ropivacaine 0.75 % without epinephrine, *ROPI-EPI* popliteal block with 30 mL ropivacaine 0.75 % with epinephrine 5 μg/mL. Values are proportions, mean (SD) or actual numbers. Differences between the groups were not significant. *Patients with medial incision beside lateral incision

Due to OR logistics, block onset could not be measured at 45 min in all patients. In case a patient did not have a complete block before the beginning of surgery, block success or failure was defined as absence or presence of pain requiring therapy in the distribution of the sciatic nerve upon arrival at the recovery directly postoperatively. Patients with a failed block were excluded and replaced, patients with a successful block remained in study. Table 2 shows sensory and motor block onset scores for patients with a successful block upon arrival at the recovery.

**Table 2:** Sensory and motor nerve block at beginning of surgery

Nerve	ROPI			ROPI-EPI		
	Complete	Partial	Absent	Complete	Partial	Absent
Tibial sensory	4	11	1	9	4	2
Tibial motor	10	3	2	9	5	1
Peroneal sensory	12	3	-	11	4	-
Peroneal motor	11	2	2	10	3	2

Groups as defined in Table 1. Values represent numbers of patients

**Fig. 2:**
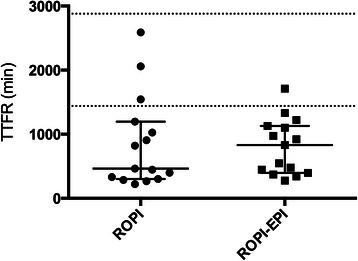
Individual data points of time to first request for postoperative analgesia. ROPI: popliteal block with 30 mL ropivacaine 0.75 % without epinephrine; ROPI-EPI: popliteal block with 30 mL ropivacaine 0.75 % with epinephrine 5 μg/mL; TTFR: Time To First Request for postoperative analgesia. Horizontal lines represent medians ± interquartile range. Dotted lines represent t = 24 and 48 h

Figure 2 shows individual TTFR data points for both groups. Median [IQR] time to first request for postoperative analgesia was 463 [300–1197] min and 830 [397–1128] min for the ROPI vs. ROPI-EPI group respectively. Hodges Lehman median difference between groups was 71 min (95 % CI −415 – 473) for the ROPI-EPI vs. ROPI group. There were no differences in any clinical outcome measures between the groups (Table 3).

**Table 3:** Clinical outcome measures

	ROPI	ROPI-EPI	*p*-value
TTFR (min)	463 [300–1197]	830 [397–1128]	0.56
NRS rest at t = 24 h	1 [0–3]	1 [1–3]	0.70
NRS movement at t = 24 h	1.5 [0–3]	2 [1–3]	0.47
NRS max during 24 h	4 [2–7]	6 [3–8]	0.17
NRS satisfaction with block	8 [8–9]	9 [8–10]	0.08

Groups as defined in Table 1. Values are median [IQR]. *TTFR* Time To First Request for postoperative analgesia, *NRS* Numeric Rating Scores (0–10)

## Discussion

The results of this study did not show a significant increase in the duration of postoperative analgesia by adding epinephrine to ropivacaine for popliteal nerve block.

A prolonged duration of postoperative analgesia by adding epinephrine to ropivacaine was expected based on our clinical experience. In a previous study, we found an indication of prolonged TTFR after the addition of epinephrine 5 μg/mL to 450 mg ropivacaine for combined sciatic/femoral nerve block for anterior cruciate ligament reconstruction [11]. However this study was underpowered to make comparisons in TTFR. In the present study we were unable to confirm this expected difference in sensory block duration. Although the difference in the median TTFR between the groups is large (367 min), the data show a large variation and data in group ROPI are skewed with a long tail and therefore are not normally distributed. The Hodges Lehman estimate of the median difference was 71 min (95 % CI −415 – 473). As a result of the large variation in our data the risk of a type II error is considerable. The absence of a statistically significant difference should therefore be interpreted with caution.

Our results are consistent with previous findings by Cederholm [9] and Weber [10]. Cederholm found no difference in duration of sensory block during epidural analgesia with 20 mL ropivacaine 0.5 % or 0.75 % either with or without epinephrine 5 μg/mL. Weber did not find an effect of epinephrine 5 μg/mL added to 20 mL ropivacaine 0.5 % and 0.2 % on postoperative analgesia via a femoral catheter after total knee replacement.

Epinephrine is thought to prolong block duration based on a decrease in local anaesthetic absorption due to local vasoconstriction at the site of injection [3]. Ropivacaine has intrinsic vasoconstrictive properties. Cederholm [13] found an inverse dose–response relationship in which the weakest solutions of ropivacaine (0.063 % and 0.125 %) showed the most marked reduction in skin blood flow measured by laser doppler as compared to normal saline. Kopacz [14] found that subcutaneous injection of ropivacaine 0.25 % and 0.75 % reduced cutaneous blood flow in pigs to a similar degree at both concentrations also measured by laser doppler. The addition of epinephrine to ropivacaine did not alter the maximum decrease in blood flow observed; however epinephrine significantly decreased blood flow when added to saline.

This quality of ropivacaine may also explain the absence of a clinical significant difference in block duration of ropivacaine either with or without epinephrine found in our study.

Our study has several limitations. We did not measure block duration by assessing sensory and motor block at regular time intervals, and TTFR is a subjective measure of block duration. However, pin-prick assessments of sensory block during 24-48 h, including night-time, is bothersome for patients and, for instance, in the present study impossible due to the post-operative cast management. From a clinical perspective duration of analgesia is more important than duration of sensory block. We therefore feel that using the TTFR as a tool to measure block duration is acceptable. Our decision to replace the two patients (one in each group) who made no request for pain relief during 48 h and in whom there were no longer signs of sensory sciatic nerve block is debatable, as it may be argued that both patients had a successful sciatic nerve block. However, since the primary outcome parameter (TTFR) was absent in these patients and the sciatic nerve block had worn off, we felt it would have been inappropriate to censor these data to 48 h and include them in the analysis.

Furthermore, anesthesiologists were not blinded for treatment allocation. All blocks were performed by experienced anesthesiologists in a standardized fashion as described in the treatment protocol. Since they were not involved in block assessment or in any other way in the conduction of the study, we believe that the absence of blinding of the anesthesiologists performing the blocks does not affect the results.

Another limitation is that we studied the duration of analgesia of the popliteal nerve block for ankle fusion surgery while the cutaneous sensory supply of the medial malleolus is by the saphenous nerve. Clendenen and Whalen histologically verified that the saphenous nerve innervates not only the skin, but also the periosteum of the medial malleolus and joint capsule [15]. Because we performed only a single-shot femoral or saphenous nerve block with mepivacaine, the TTFR in individual patients may have been triggered by pain in the distribution of the saphenous nerve, and thus reflect the duration of sensory block of the femoral or saphenous nerve rather than the sciatic nerve. Standard incision for ankle fusion and subtalar fusion is on the lateral side. However, an additional medial incision was made in 5/30 patients. In four of these patients the TTFR was > 360 min. Because the duration of sensory femoral or saphenous nerve block is shorter, we believe that the effect of pain in the distribution of the saphenous nerve on the TTFR is minimal.

## Conclusions

In conclusion, we were unable confirm an expected difference in the duration of postoperative analgesia by adding epinephrine to ropivacaine for popliteal nerve block. This may be explained on the basis of the intrinsic vasoconstrictive properties of ropivacaine or due to a large variation in the individual TTFR, the absence of a significant difference may also be caused by a type II error.
